# The atypical homeoprotein Pbx1a participates in the axonal pathfinding of mesencephalic dopaminergic neurons

**DOI:** 10.1186/1749-8104-7-24

**Published:** 2012-07-02

**Authors:** Paola Sgadò, Elisabetta Ferretti, Daniel Grbec, Yuri Bozzi, Horst H Simon

**Affiliations:** 1 Laboratory of Molecular Neuropathology, Centre for Integrative Biology (CIBIO), University of Trento, Trento, Italy; 2 Interdisciplinary Center of Neuroscience, Department of Neuroanatomy, University of Heidelberg, Heidelberg, Germany; 3 Department of Cell and Developmental Biology, Weill Medical College of Cornell University, New York, NY, USA; 4 Neuroscience Institute, National Research Council (CNR), Pisa, Italy

**Keywords:** Axonal outgrowth, neurodegenerative disease, Prep1, substantia nigra, transcription factors, ventral tegmentum

## Abstract

**Background:**

The *pre B-cell leukemia transcription factor 1 (Pbx1)* genes belong to the three amino acid loop extension family of homeodomain proteins that form hetero-oligomeric complexes with other homeodomain transcription factors, thereby modulating target specificity, DNA binding affinity and transcriptional activity of their molecular associates.

**Results:**

Here, we provide evidence that *Pbx1* is expressed in mesencephalic dopaminergic neurons from embryonic day 11 into adulthood and determines some of the cellular properties of this neuronal population. In *Pbx1*-deficient mice, the mesencephalic dopaminergic axons stall during mid-gestation at the border between di- and telencephalon before entering the ganglionic eminence, leading to a loose organization of the axonal bundle and partial misrouting. In *Pbx1*-deficient dopaminergic neurons, the high affinity netrin-1 receptor, deleted in colon cancer (DCC), is down-regulated. Interestingly, we found several conserved Pbx1 binding sites in the first intron of *DCC*, suggesting a direct regulation of *DCC* transcription by Pbx1.

**Conclusions:**

The expression of *Pbx1* in dopaminergic neurons and its regulation of *DCC* expression make it an important player in defining the axonal guidance of the midbrain dopaminergic neurons, with possible implications for the normal physiology of the nigro-striatal system as well as processes related to the degeneration of neurons during the course of Parkinson’s disease.

## Background

*Pre B-cell leukemia transcription factor 1* (*Pbx1*) encodes a transcription factor, belonging to the PBC (Pbx1 to 4) subclass of the three amino acid loop extension (TALE) proteins characterized by an atypical homeodomain [1]. Studies of the Pbx proteins and their *Drosophila* homolog *Extradenticle (exd)* revealed that they form stable complexes with other homeodomain transcription factors, such as *Hox* and *Engrailed*, as well as other non-homeodomain proteins [2]. The interaction with Pbx modulates the target selectivity, the DNA binding affinity and the transcriptional activity of the associated homeoproteins [3]. An example of the modulation of transcriptional activity by the Pbx transcription factors is the regulation of *Fibroblast growth factor 8* (*Fgf8*) expression by the Engrailed transcription factors. A highly conserved region in the large intron of the *Fgf8* gene contains an Engrailed/Pbx binding site. This part of the enhancer increases transcriptional activity by three to fourfold in the presence of embryonic nuclear extract containing the Engrailed proteins and Pbx1, and point mutations in the binding site inactivate it [4].

*Pbx* loss of function phenotype is to a large part a reflection of the phenotypic alterations observed after functional ablation of the associated molecular partner. In *Drosophila*, for example, embryos lacking *exd* (zygotic or maternal) show the typical homeotic transformations in the thoracic and abdominal segments that resemble the loss of function phenotypes of the *Hox* genes cooperating with *exd*, although their expression is unaltered [5]. In mammals, the correlation between phenotypes of mutants deficient for *Pbx1* and null mutants for the molecular partners is not as evident [6]. *Pbx* genes have been implicated in development of the skeleton [7], pancreas [8], kidney, adrenal glands [9,10], thymus [11], spleen [12] and in hematopoiesis [13]. A number of homeodomain transcription factors play a major role in the development of all of these tissues and organs systems and may act as cofactors for *Pbx* genes.

We have previously demonstrated that the *Engrailed* genes are required for survival of the mesencephalic dopaminergic (mesDA) neurons [14,15]. The survival function of *Engrailed* genes is unique to these neurons and is not shared with other neuronal populations expressing the genes, like the cerebellar granule cells [16,17] or the V1 interneurons in the spinal cord [18]. The cooperative binding to Pbx1 protein has already been shown to modulate the regulation of *Fgf8* expression by the *Engrailed* genes, a crucial factor for the development of the mesDA neurons [4,19,20]. We therefore hypothesized that cooperative binding to Pbx proteins may modulate the target selectivity of the *Engrailed* genes in mesDA neurons.

We examined the expression and function of the *Pbx* genes in this neuronal population during development and show here that a splicing variant of *Pbx1Pbx1a*, and one of the *Prep* genes, *Prep1*, are expressed by mesDA neurons. Furthermore, our analysis of *Pbx1* mutant mice demonstrates a role of *Pbx1* in axon guidance through the regulation of the netrin-1 receptor, *deleted in colon cancer* (*DCC*). Interestingly, despite increasing evidence of a cooperative function of Engrailed and Pbx transcription factors in vertebrates development [4,21], we could not find a 1:1 correlation between *Engrailed* and *Pbx1* mutants’ phenotype. In our case, a more detailed analysis of different single and compound mutants might be necessary to confirm our original hypothesis of a Pbx/Engrailed functional cooperative binding playing a significant role in the development and survival of mesDA.

## Results

### *Pbx1* expression in mesencephalic dopaminergic neurons

**Figure 1:**
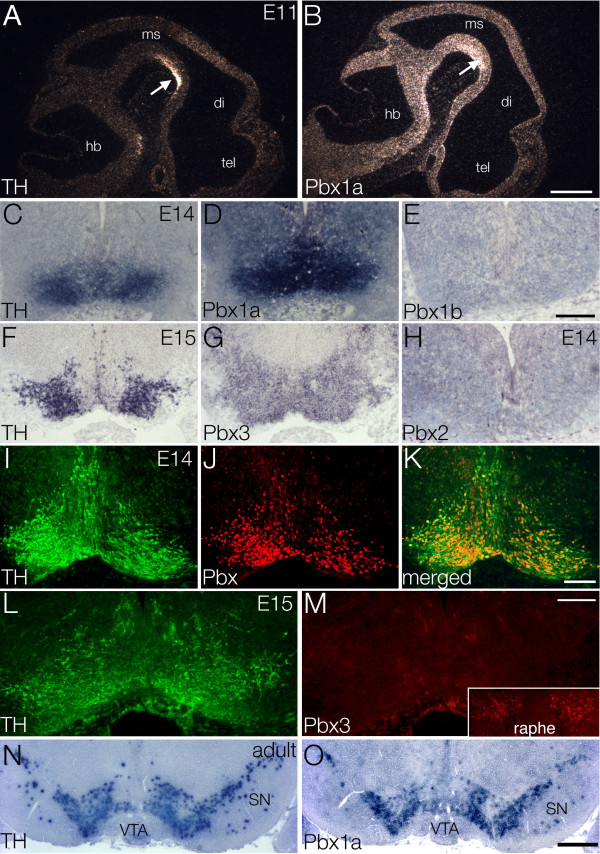
**Expression of Pbx1a in mesencephalic dopaminergic neurons. ***In situ* hybridization using ^35^ S- (A, B) and digoxigenin-riboprobes (C-H, N, O) against *TH* (A, C, F, N), *Pbx1a* (B, D, O), *Pbx1b* (E), *Pbx2* (H) and *Pbx3* (G) on E11 sagittal (A, B) and coronal sections of E14 (C, D, E, H), E15 (F,G) and adult (N, O) mice. Fluorescent double labeling on coronal sections of E14 (I-K) and E15 (L, M) embryos using antibodies against TH (I, L) pan-Pbx (J) and Pbx3 (M). (**A, B**) At E11, *TH* expression is confined to mesDA neurons on the pial surface of the ventral mesencephalon (arrow) (A). The parallel section reveals the full extent of the *Pbx1a* expression with an elevated signal at the position of mesDA neurons (arrow) (B). (**C-H**) *Pbx* members’ expression in the ventral midbrain. *Pbx1a* (D) co-localizes with *TH* (C). *Pbx1b* (E) and *Pbx2* (H) are not detectable in the ventral midbrain whereas *Pbx3* (G) shows a diffused signal partially overlapping with *TH* (F). (**I-K**) Immunohistochemical double labeling using TH (I, green) and pan-Pbx (J, red) antibodies reveals the co-expression of Pbx1 and TH in mesDA neurons (K). (**L, M**) Double immunohistochemistry using TH (L, green), and Pbx3 (M, red) antibodies shows no detectable Pbx3 protein in the ventral midbrain. Insert: Pbx3 positive staining in raphe nucleus. (**N, O**) Expression of *Pbx1a* in the adult co-localizes with *TH* in ventral tegmental (VTA) and nigral (SN) dopaminergic neurons. Telencephalon (tel), diencephalon (di), mesencephalon (ms), hindbrain (hb). Dorsal to the top, A and B rostral to the right. Scale bars A-B, N, O = 500 μm, C-H, = 200 μm, and I-M = 100 μm. mesDA: mesencephalic dopaminergic.

**Figure 2:**
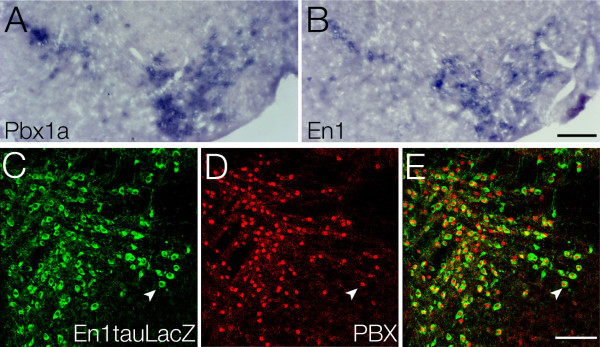
**Adult expression of Pbx1a and En1 in mesencephalic dopaminergic neurons. ***In situ* hybridization using digoxigenin-labeled riboprobes against (**A**) *Pbx1a* and (**B**) *En1* on adult coronal sections and immunohistochemistry on adult mid-sagittal sections of *En1+/tlZ* mice using antibodies against (**C**) pan-Pbx and (**D**) ß-galactosidase to reveal the En1tauLacZ reporter gene. (A, B) *Pbx1a* and *En1* expression co-localize in adult mesDA neurons. (C-E) Pbx1 protein (D, red) is detectable in the nucleus of all En1tauLacZ-expressing (C, green) mesDA neurons (E, arrowheads). Scale bars A, B = 250 μm and C-E = 200 μm . mesDA: mesencephalic dopaminergic.

To examine expression of the *Pbx* family members in mesDA neurons, we performed *in situ* hybridization on midbrain sections of various ages. We restricted our analysis to those family members, *Pbx12* and *3*, which are expressed in the brain [22]. Of note, only *Pbx1a*, a splicing variant of *Pbx1,* co-localized with *tyrosine hydroxylase* (*TH*), the key enzyme of dopamine synthesis (Figure 1). At embryonic day (E) 11, *Pbx1a* was expressed in the entire developing midbrain neuroepithelium. Ventrally on the pial site, an elevated signal was observable that overlapped with TH in the parallel section, suggesting co-expression (Figure 1A, B). At E14, Pbx1a expression was more restricted and now clearly overlapped with TH (Figure 1C, D). The staining with a pan-Pbx antibody at the same embryonic stage revealed that each TH-positive cell body in the midbrain possessed a Pbx1-positive nucleus (Figure 1I-K). At postnatal ages, *Pbx1a* expression was decreased in intensity and disappeared in many brain regions, but remained at high levels in all mesDA neurons (for the adult see Figure 1N, O). Furthermore, a double immunostaining using the pan-Pbx antibody and an antibody against β-galactosidase to detect the Engrailed1 (En1) reporter LacZ [23] revealed that Pbx1a is co-expressed with En1 in these neurons (for the adult see Figure 2A-E). We also detected by *in situ* hybridization a diffuse *Pbx3* RNA signal in the ventral midbrain from E14 into the adult (Figure 1G), but a Pbx3 specific antibody on wild type was unable to detect any Pbx3 protein in mesDA neurons (Figure 1L, M). However, Pbx3 protein was detectable in other brain regions, like for example the raphe nucleus (Figure 1L, M insert).

### Pbx1 sub-cellular localization

The activity of PBC proteins is in part regulated by nuclear import, which is mediated by dimerization with homeoproteins of the MEINOX (MEIS and KNOX) sub-class, or by phosphorylation [24-27]. The MEIS subfamily of TALE proteins includes the products of the vertebrate *Meis1-3*, while the PREP subfamily includes the vertebrate *Prep1* and *Prep2*. Exd and Pbx proteins have been shown to require MEIS/PREP for their nuclear import in specific cell contexts, such as limb mesenchymal cells in vertebrates or limb imaginal disc cells in flies [28-32].

**Figure 3:**
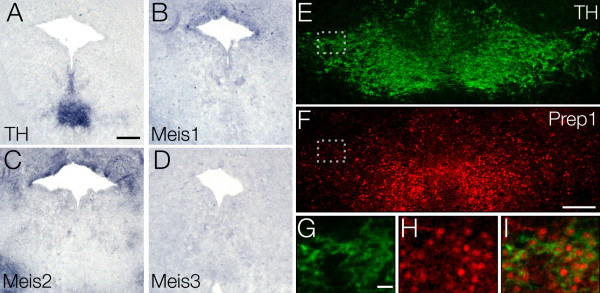
**Expression of MEINOX genes in ventral midbrain. ***In situ* hybridization using digoxigenin-labeled riboprobes against (**A**) *TH*, (**B**) *Meis1*, **(C)***Meis2* and (**D**) *Meis3*. Immunohistochemistry using antibodies against TH (E, G) and Prep1 (F, H). (A-D) None of the *Meis* family members is expressed by E15 mesDA neurons. (**E**,**F**) TH (E) and Prep1 (F) are co-expressed in mesDA neurons. (**G-I**) Magnification of dashed box, showing Prep1-positive nuclei in TH-positive cell body. Dorsal to the top. Scale bars, A-D = 200 μm, E, F = 250 μm, G-H = 50 μm. mesDA: mesencephalic dopaminergic.

Since the Pbx1a protein was localized in the nucleus of mesDA neurons, we investigated whether any of the *Meis* genes are also expressed in these neurons. We found *Meis1, Meis2* and *Meis3* expression in telencephalon, diencephalon, midbrain and hindbrain as previously described [33], but none of them in mesDA neurons (Figure 3A-D). Despite previous reports of a ubiquitous expression of *Prep1* in the developing brain from as early as E7.5, we detected by immunohistochemistry a specific Prep1 domain in the ventral midbrain, co-localized with TH (Figure 3E-I), indicating that the nuclear transport of the Pbx1 protein is likely achieved in this neuronal population by molecular association with Prep1.

### Analysis of *Pbx1* mutant mice

**Figure 4:**
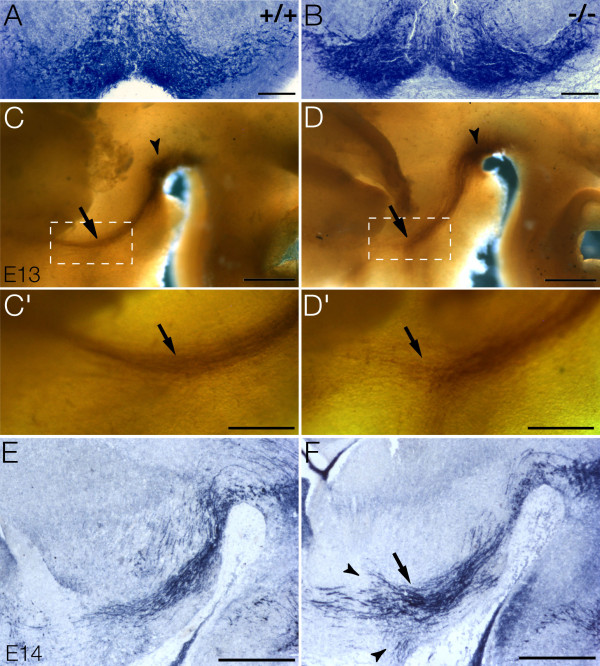
**Misrouting of dopaminergic axons in Pbx1-deficient embryos.** TH immunohistochemistry on E15 coronal (A, B) and E14 sagittal sections (E, F), and on E13 dissected brains in whole mount preparation (C-D’) of wild type (A, C, E) and *Pbx1−/−* mutant embryos (B, D. F). (**A, B**) Distribution of mesDA neurons in the ventral midbrain is identical in wild type and *Pbx1−/−* mice. (**C-D’**) E13 whole mount preparation of isolated neural tube. TH-positive neurons are located in the ventral midbrain (arrowheads) of wild type (C) and *Pbx1*-deficient (D) embryos. Wild type TH-positive axons have reached deep into the ventral telencephalon, whereas the *Pbx1−/−* axons have prematurely stopped their growth prior to entry of the ganglionic eminence and begin to defasciculate at the tip (arrow) (D, D’). C’ and D’ are higher magnification of the dashed box in C and D. (**E, F**) At E14, the bundle of TH-positive axons is compact, fasciculated and directed towards the ventral telencephalon in the wild type (E). In the mutants, the axon bundle is wider and disorganized (arrow) and shows misrouted axonal tips (arrowheads) (F). Dorsal to the top, C-F rostral to the left. Scale bars A, B = 200 μm, C, D, E, F = 500 μm and C’, D’ = 250 μm. mesDA: mesencephalic dopaminergic.

In order to investigate the role of *Pbx1a* in mesDA neurons, we analyzed homologous recombinant mutant mice null for *Pbx1*[7]. Up to E15.5, when the mutant mice die, mid/hindbrain morphology and the distribution of mesDA neurons appeared normal (Figure 4A, B); however, the mutants showed aberrant mesDA axonal projections. In the E13 whole mount preparations, the wild type mesDA neurons extended their axons deep into the ganglionic eminence [34] (Figure 4C, C’), whereas in *Pbx1* null mutants, the DA axons stopped growing at the border between tel- and diencephalon, and defasciculated (Figure 4D, D’). One day later, at E14, some of the *Pbx1*-deficient mesDA axons reached into the ganglionic eminence (data not shown) but the axonal bundle was loosely packed and a small part of the axons had misrouted at the same position in the ventral forebrain where they had stalled at E13 (Figure 4E, F).

**Figure 5:**
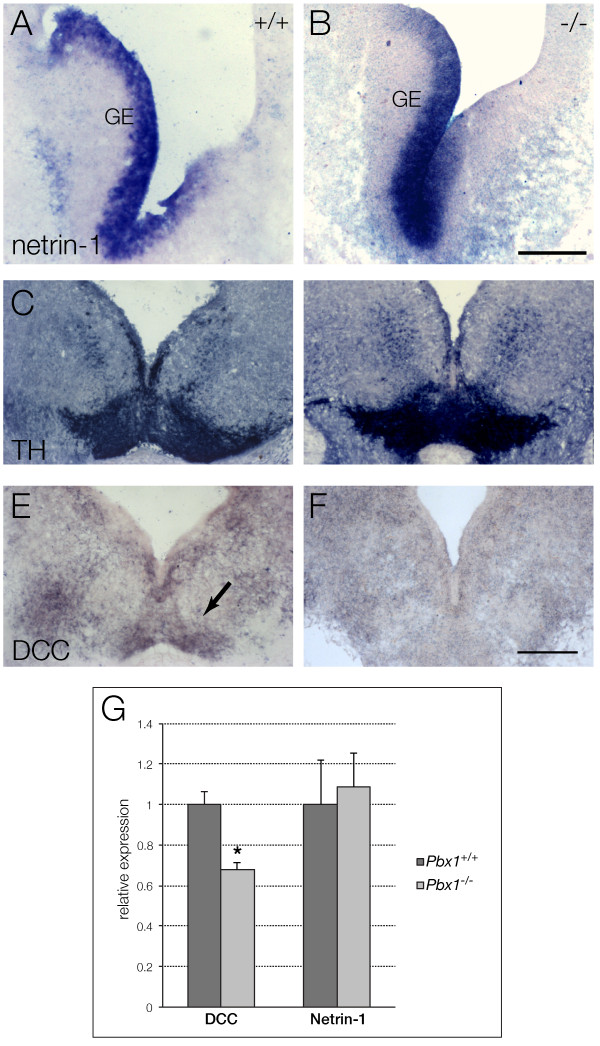
**Netrin-1 and DCC expression in Pbx1 null and wild type embryos. ***In situ* hybridization using digoxigenin-labeled riboprobes against *netrin-1* (A, B) and *DCC* (E, F), immunohistochemistry with antibodies against TH (C, D) on E14 mouse coronal section of wild type (A, C, E) and *Pbx1−/−* mutants (B, D, F) at level of midbrain. **(A, B)** Identical expression of *netrin-1* in the ganglionic eminence (GE) of wild type (A) and *Pbx1*-deficient (B) embryos. **(C-F)** The parallel TH and DCC stained sections reveal *DCC* expression in wild type mesDA neurons, but no expression in *Pbx1*-deficient embryos at the same level. Dorsal to the top. Scale bars = 300 μm. **(G)** Quantitative PCR using *DCC-* and *netrin-1*- specific primers and probes on mRNA extracts from E13.5 ventral midbrain from *Pbx1+/+* and *Pbx1−/−* mutants. Expression levels are relative to the housekeeping gene *RLPL0*, values are expressed as 2^-ΔCt^ mean ± standard error of the mean; n ≥ 4 for each experiment(*P <0.05). mesDA: mesencephalic dopaminergic.

Recent studies indicated a role of *netrin-1/DCC* signaling in the guidance of mesDA axons [35,36], thus, we investigated *Pbx1* mutant mice for alterations in the expression of *netrin-1* and its high affinity receptor *DCC*[37]. Despite the widespread expression of *Pbx1* in the telencephalon, *netrin-1* expression in the basal ganglia of E14 *Pbx1* mutant appeared normal (Figure 5A, B); instead, expression of its receptor *DCC*[38] was absent in *Pbx1*-deficient mesDA neurons (Figure 5E, F) identified by TH immunohistochemistry on the parallel sections (Figure 5C, D). To confirm the absence of DCC in mesDA neurons we performed quantitative PCR on E13 ventral midbrain tissue of *Pbx1* mutant mice compared to littermate controls. We found a reduction of approximately 35-40 % in the expression of *DCC* in the entire ventral midbrain of *Pbx1* mutant mice while the expression of *netrin-1* was unaltered (Figure 5G).

### Analysis of mesencephalic dopaminergic markers in *Pbx1* mutants

In order to assess whether *Pbx1* deletion leads to the altered expression of other genes associated with mesDA neurons phenotype, concomitantly with the perturbation observed in *DCC* gene expression, we performed *in situ* hybridization on E15 embryos using probes specific for *Nuclear receptor related 1 (Nurr1)*[39], *En1, En2*[40], *LIM homeobox transcription factor 1-beta (Lmx1b)*[41], *Pituitary homoebox 3 (Pitx3)*[42], *THDopamine transporter (DAT)Dopa decarboxylase (AADC)Dopamine receptor 2 (DRD2)Ret oncogene (c-ret)Glial cell line-derived neurotrophic factor family receptor alpha 1 (GFR-α1)*[43], *Aldehyde dehydrogenase family 1 subfamily A1 (Ahd2)*[44] and *α-synuclein*[40]. None of them were altered in *Pbx1−/−* mutants.

Conversely, the absence of *DCC* expression in *Pbx1*-deficient mutant embryos suggested that DCC is a direct target of Pbx1. In order to investigate this hypothesis, we searched for putative Pbx1 binding sites in *DCC* regulatory region by *in silico* analysis. Our syntenic alignment of human, rat and mouse genomic sequences 20 kb upstream and 10 kb downstream of the start codon of the *DCC* gene in combination with the TRANSFAC database weight matrix for the *Pbx1* consensus sequence revealed three conserved Pbx1 binding sites in the first intron of *DCC* at positions A: 775–786, 765–776, 761–772, B: 2059–2067, 2122–2130, 1996–2004 C: 2584–2592, 2645–2653, 2515–2523 bases downstream of the ATG in human, mouse and rat, respectively (data not shown). However no significant DNA enrichment was achieved by chromatin immunoprecipitation (ChIP) either with a specific Pbx1 antibody or with the pan-Pbx antibody (data not shown), indicating that regulation of *DCC* expression by Pbx1 is probably not direct.

## Discussion

We show here that *Pbx1a* is expressed in mesDA neurons from E11 into adulthood. During early embryogenesis, its expression in the neural tube is abundant, and becomes later confined in the ventral midbrain to only mesDA neurons. The co-expression of *Pbx1a* and *Prep1* in mesDA neurons suggests that Pbx1 nuclear localization is achieved in this neuronal population through molecular association with Prep1. We, furthermore, show an aberrant mesDA axonal projection in *Pbx1−/−* embryos, which is likely the result of the loss of *DCC* expression. However we were not able to demonstrate direct Pbx1 binding on the three highly conserved Pbx1 binding sites in the first intron of *DCC* by ChIP.

A number of studies have shown molecular interactions between Pbx proteins and several other transcription factors and transcriptional co-regulators. The most studied Pbx partners are the Hox proteins. However, Pbx members form functional heterodimeric complexes with other homeoproteins, such as Engrailed and Pdx1, and other non-homeodomain transcription factors of the basic helix-loop-helix, forkhead and Smad family, as well as with members of the nuclear receptor superfamily [2,6]. Pbx loss of function phenotype is very often correlated to the function of the associated partner. *Pbx1*-deficient mice die at E15.5, displaying severe hypoplasia (lungs, liver, stomach, gut, kidneys and pancreas), ectopia (thymus and kidneys) or aplasia (spleen, adrenal gland) of multiple organs, and widespread defects of the axial and appendicular skeleton [7]. Although mice with *Pbx1* targeted mutation exhibit some degree of homeotic transformations, they do not perfectly resemble mutants for *Hox* genes, their most studied partners. The same can be said for other Pbx mutants. *Pbx3*-deficient mice survive to term, but die soon after birth from central respiratory failure [45]. *Pbx1* and *Pbx3* have overlapping embryonic expression domains and could therefore exhibit redundant functions. In contrast to *Pbx1*- and *Pbx3*-deficient mice, *Pbx2*-deficient mice are viable and display no apparent phenotype despite its broad expression [46]. Therefore the phenotype of the Pbx targeted mutants could be the result of compensatory functions of other Pbx members and/or partial partner-independent functions [2,6].

The phenotypical alterations in mesDA neurons of *Pbx1*-deficient mice can be considered in correlation to the well-described *Engrailed* phenotype in these cells. The targeted deletion of both *Engrailed* genes leads to severe tissue deletion in the mesencephalon and loss of mesDA neurons at birth [40]. A more detailed analysis of these mutant mice revealed that the dopaminergic neurons are generated in the mesencephalic flexure, but die by E14 without extending axonal processes [15]. MesDA neurons in *Pbx1*-deficent embryos survive beyond E14 and are able to extend axons; a phenotype that seems to diverge from the complete ablation of mesDA neurons observed in *Engrailed* double mutant embryos. Yet a cooperative function of Engrailed and Pbx1 cannot be excluded on the base of this sole phenotypic resemblance. *Engrailed* mutation show a gene-dose dependent effect on the survival of mesDA neurons [40] and no information have been reported about the axonal projections of mesDA neurons in other single or compound *Engrailed* mutants*.* Furthermore, our analysis does not exclude a redundant effect of other *Pbx* genes. The presence of *Pbx3* mRNA expression in these neurons indicates the possibility of a compensatory effect in absence of *Pbx1*, therefore restoring the threshold Pbx proteins concentration required for a correct development.

We report here that *Pbx1* loss of function leads to defasciculation and misrouting of mesDA axons in the border between di- and telencephalon. Since *Pbx1* is expressed in mesDA neurons as well as in the developing target tissue [47], the axonal outgrowth phenotype of *Pbx1*-deficient mice could reflect alterations in either of the two. The unaltered expression of *netrin-1* in the ganglionic eminence, the intact morphology of the tissue and the loss of *DCC* expression suggest that the mesDA axonal phenotype is likely attributable to a cell-autonomous function of Pbx1.

Several studies suggest that multiple cues collaborate to guide dopaminergic axons into a restricted domain through the diencephalon. Initially, migration of mesDA axons rostrally is determined by repulsion from a posterior source of semaphorin. Once in the diencephalon, mesDA axons are constrained in a narrow path established by multiple signals that keep axons from diverging ventrally or dorsally. The ventral boundary requires both Robo/Slit [48,49] and Netrin/DCC [35,36] opposing actions, as both slits repulsion and netrins attraction actions contribute to prevent dopaminergic axons from crossing the midline. Dorsal repulsion instead is likely mediated by attractive cues only, such as netrin and Sonic hedgehog [35,49-51]. Finally, mesDA projections into the basal forebrain and cortex require an unusual attractive activity of semaphorin [52].

A recent analysis of *DCC* loss of function *in vitro* and *in vivo* demonstrated that *DCC* regulates neuronal precursor cell migration, axon guidance and axonal terminal arborization [36]. Nevertheless, even in absence of *DCC* expression, mesDA axons are able to reach their target tissue [36]. Differently from the previous report, however, in *Pbx1*-deficient embryos, loss of *DCC* expression has no effect on cell migration and seems to affect only long-range axon guidance. In *Pbx1*-deficent mice, axonal outgrowth is not affected until the mesDA neurons reach the border region between di- and telencephalon, and only at this point does *Pbx1*-mediated DCC/netrin signaling seem to be required. Unfortunately, *Pbx1* mutant mice die at E15.5, preventing further analysis of the phenotype induced by the loss of *DCC* expression in these mice. No information is available on the embryonic phenotype of *DCC* mutants to be compared with those of *Pbx1* mutants. Furthermore, analysis at later stages of the basal forebrain structures affected by abnormal nigro-striatal axonal targeting (dorsal striatum, olfactory tubercle, etc…) is not possible in *Pbx1*-mutants as complete maturation of dopaminergic innervations to the forebrain takes place between E15 and P0 [34,53].

According to the Stein and Tessier-Lavigne ‘Hierarchical organization of guidance receptors’ model [54], activation of DCC by netrin, and concomitantly of Robo by Slit, leads to silencing of the attractive DCC-mediated netrin response without affecting its growth-stimulatory effect. Indeed, both *DCC* and *Robo* are expressed in mesDA neurons at developmental stages consistent with the defect observed in *Pbx1* mutant embryos and could contribute to the observed phenotype [35,36,49]. Furthermore, a recent study indicated that loss of Slit/Robo signaling leads to widespread errors in mesDA axonal trajectories in the diencephalon, similar to those observed in *Pbx1*-deficient mice [49].

## Conclusions

In this study, we show that Pbx1 and possibly its co-factor Prep1 are part of the transcriptional factor network that control a key step in mesDA neuronal differentiation by regulating the establishment of mesencephalic-striatal axonal projection. The axon guidance pathways are not just important in development of mesDA neurons they may regulate survival of this neuronal population throughout life, as suggested by genetic linkage studies and their connection to sporadic Parkinson’s disease [55,56]. Therefore, *Pbx1* may be important in determining the vulnerability of mesDA neurons to degeneration during the early phases of Parkinson’s disease.

## Methods

### Mutant mice

Targeted mutation of *Pbx1* and *En1tauLacZ* mice has previously been described [7,23]. *Pbx1+/−* and *En1+/tlZ* adult mice were crossed into a C57/Bl6 background. The colony was maintained at the central animal facility at the University of Heidelberg. Experiments were carried out in accordance with the European Communities Council Directive of 24 November 1986 (86/609/EEC) for the care and use of experimental animals; all procedures were approved by the central animal facility at the University of Heidelberg. Each of the described phenotypes was found in all analyzed mutant animals (n ≥ 4).

### *In situ* hybridization

Radioactive and digoxigenin *in situ* hybridizations have been previously described [40]. The riboprobes corresponded to 1644 to 2277 of NM_183355 (*Pbx1a*), 1917 to 3049 of NM_008783 (*Pbx1b*), 1468 to 2264 of NM_017463 (*Pbx2*), 1665 to 2331 of NM_016768 (*Pbx3*) and 2780 to 3304 of NM_007831 (*DCC*). *TH* and *En1* are described elsewhere [40].

### Immunohistochemistry

All immunohistochemistry, including the whole mount staining, was performed as described [40] using rabbit and sheep anti-TH antibodies (AB152 and AB1542 EMD Millipore Inc., Billerica, MA, USA) at 1:1,000, rabbit anti-pan-Pbx antibody (sc-888 Santa Cruz Biotechnology Inc., Santa Cruz, California, USA) at 1:2,000, rabbit anti-Pbx3 antibody (sc-891 Santa Cruz Santa Cruz Biotechnology Inc., Santa Cruz, California, USA) at 1:1,000, goat anti-ß-galactosidase at 1:10,000 (Arnel Products Co., New York, NY, USA) and mouse anti-Prep1 antibody at 1:200 (05–766 EMD Millipore Inc., Billerica, MA, USA). The pan-Pbx antibody recognizes a common C-terminal peptide in all of the 50 kDa splice variants of Pbx1, Pbx-2 and Pbx3.

### Real time PCR

Quantitative PCRs were performed with a Biorad CFX384 system by using preformulated TaqMan Gene expression assays (Invitrogen, Life Technologies Inc., Carlsbad, California, USA) and calculating the results with the comparative Ct method. The assays had the following identification tags: Mm00514509_m1 (*DCC*), Mm00500896_m1 (*netrin-1*) and Mm01974474_gH (*RPLP0*). Dissection of ventral midbrain tissue has been previously described [15]. The dissected ventral midbrains were homogenized, the RNA isolated using the RNeasy Mini kit (Qiagen group, USA) and reverse-transcribed using the VILO Superscript cDNA synthesis kit (Invitrogen, Life Technologies Inc., Carlsbad, California, USA). Each individual PCR was done in three biological replicates.

### *In silico* promoter analysis

Syntenic alignment and analysis of transcription factor binding sites of genomic sequences was performed using ECR Browser and rVista2.0 software (http://www.dcode.org/). For the identification of transcription factor binding sites, rVista2.0 uses a recently developed method, which combines ‘suffix tree’-based fast subsequence search with position weight matrices.

## Abbreviations

ChIP, chromatin immunoprecipitation; DCC, Deleted in colorectal cancer; E, embryonic day; Exd, Extradenticle; Fgf8, Fibroblast growth factor 8; mesDA, mesencephalic dopaminergic; MEINOX, MEIS and KNOX subclass of the TALE superclass; PBC, PBC domain family of the TALE superclass; Pbx, Pre B-cell leukemia homeobox; PCR, polymerase chain reaction; TALE, three amino acid loop extension; TH, tyrosine hydroxylase.

## Competing interests

The authors declare that they have no competing interests.

## Authors’ contributions

PS designed and carried out all the experiments, analyzed and interpreted the data, and drafted the final version of the manuscript. EF and DG provided some experiments, and helped with interpretation of the data and writing of the manuscript. YB helped with interpretation of the data and revised the manuscript. HHS conceived of the study, participated in its design and coordination and drafted the manuscript. All authors read and approved the final manuscript.
